# Expressive instructions: ethnographic insights into the creativity and improvisation entailed in teaching physical skills to medical students

**DOI:** 10.1007/s40037-018-0446-5

**Published:** 2018-07-27

**Authors:** Anna Harris, Jan-Joost Rethans

**Affiliations:** 1 Faculty of Arts and Social Sciences, Maastricht University, Maastricht, The Netherlands; 2 Faculty of Health, Medicine and Life Sciences, Maastricht University, Maastricht, The Netherlands

**Keywords:** Skill, Expertise, Qualitative research

## Abstract

**Introduction:**

Creativity and improvisation are recognized as important aspects of training expertise in domains such as business and the arts, yet rarely discussed in medical education. This article examines how creativity and improvisation play out in the ways teachers give ‘expressive instructions’ to medical students when teaching physical skills.

**Methods:**

Ethnographic fieldwork was conducted in a medical school in Maastricht, the Netherlands, with first, second and third year students learning physical examination skills. Over 230 h of fieldwork was conducted in the Skills Lab, including 34 tutorials of 1.5 h duration, with 11 different teachers and over 500 students. Patterns found in the fieldnotes were thematically analyzed using an inductive approach, drawing on sociological theories of craftsmanship.

**Results:**

Findings showed that teachers improvise beyond the standardized lesson structure and classroom set-up, giving what we call, drawing on sociological theory, ‘expressive instructions’. This was visible in two main ways: 1) by teachers using their own bodies; 2) by teachers using materials that came to hand.

**Discussion:**

This research highlights the important yet underexplored role of creativity and improvisation in teaching physical skills. Creativity and improvisation appear to be particularly important when training expertise in skills that are difficult to articulate and thus require expressive instructions, due for example to their sensory nature. Focusing on how expressive instructions play out in medical education offers insights into the tacit components of expertise development, a process which builds upon a long period of teachers’ skilled practice.

**Electronic supplementary material:**

The online version of this article (10.1007/s40037-018-0446-5) contains supplementary material, which is available to authorized users.

## What this paper adds

Creativity and improvisation are recognized as important aspects of training expertise in domains such as business and the arts, yet rarely discussed in medical education. This research helps to address this gap in knowledge by examining how creativity and improvisation play out in teaching physical skills, using an ethnographic methodology to research practices that are often taken for granted. The findings of the study, which show that teachers creatively use ‘expressive instructions’ when training expertise in skills difficult to articulate, offer new, sociological and anthropological informed insights into theoretical and pedagogical understandings of expertise development in teaching physical skills.

## Introduction

When a chef or cookbook writer instructs on how to make a dish, they must not only provide a clear guide to their students, with important steps and signposts, but they must also find creative ways in which to share and teach the tacit, bodily, sensory skills entailed in cooking. In his book *The Craftsman*, the sociologist Richard Sennett considers the teaching of skills through this example of recipes [1]. He shows that how to prepare one dish, *Poulet a la d’Albufera* (stuffed chicken), can be instructed in many different ways, from Julia Child’s sympathetic illustrations to help the cook, to Elizabeth David’s scenic narrative style that draws on historical references, to his own cooking class instructor’s emphasis on metaphors. Sennett calls these different ways of sharing the recipe ‘expressive instructions’.

Teachers also need to be creative when they teach clinical skills such as physical examination. They need to find imaginative ways to describe instructions expressively in protocols and then to improvise with these scripts in class in order to share expertise with students. Yet such practices often go unnoticed in medical education; they are taken-for-granted aspects of teaching and learning. Considering the importance of creativity and improvisation in training expertise, and their documented role in other domains such as cooking [2], business [3] and science [4], as well as education more broadly [5], it seems crucial to understand more about their role in medical education. It is particularly important and pertinent to understand the role of creativity and improvisation involved in the sharing of knowledge that is hard to articulate and difficult to measure such as the tacit, bodily, sensory skills entailed in physical examination and diagnosis. By using the word ‘articulate’ here, we refer to the anthropologist Tim Ingold’s [6] understanding of the term as it applies to learning skills: that teachers may be able to tell what they know about a skill through demonstration in words or gestures, they can specify about materials and techniques explicitly, but the articulation of the sensory awareness required to do something skilfully is often elusive.

To date, the importance of creativity and improvisation in teaching has been generally overlooked in the medical education literature. Some scholars [7–10] have documented, with an element of controversy [11], the importance of creativity and improvisation in* learning*. Research has shown how students must learn to flexibly respond to patients, particularly in clinical communication [7–10]. This work has met a critical response, raising arguments that overemphasis on creativity and improvisation can be damaging to relations with patients [11]. This article contributes to this debate in medical education by considering the role of improvisation in *teaching *physical skills.

Creativity and improvisation are also controversial concepts due to the ways in which they seem, on the surface, to work against the goals of standardization in teaching and learning, and the need for defined curricula to meet learning goals. Such practices are often regarded as too subjective to warrant attention by researchers who aim to focus on objective scientific variables. Yet, as any jazz pianist would attest to, improvisation is not an easy task and requires dedication and precision [12]. In fact, being able to improvise is often a process and endpoint of a long period of skilled practice. It relies on skilled practitioners translating difficult pedagogical points using creative methods which draw from their own expertise. Education research shows that creativity and improvisation require a high degree of pedagogical content knowledge and are best suited to tasks with no clear-cut answers [5]. Drawing from a constructivist perspective, R. Keith Sawyer argues that the lens of improvisation emphasizes the interactional and responsive creativity of teachers [5].

This article approaches the topic of creativity and improvisation in teaching using a sociological approach, in particular engaging with Sennett’s work on craftsmanship [1]. Sennett is interested in skilled labour and uses a far-reaching array of examples of craftsmanship, including doctoring, to explore these practices historically and in contemporary times. One of his interests is in how skilled labour is passed on from master to apprentice. He takes the example of how guidance is given in the form of written instructions that show, rather than tell; instructions that ‘connect the technical craft to the imagination’ [1]. These are what Sennett refers to as ‘expressive instructions’. This paper focuses on the expressive instructions of teachers teaching physical skills. We show, based on ethnographic research, that expressive instructions entailing creativity and improvisation are an important element of teaching these skills to medical students.

While Sennett explores the term ‘expressive instructions’ using the example of recipes, in this article we do not focus on the most comparable written text in medical education—the physical examination teaching guide. Writing these texts certainly involves a lot of work in expressive instruction; however, we have not yet conducted the fieldwork which explores this process with teachers. Nor have we done a close textual analysis of such written forms. While these aspects of writing instructions will be explored in later research, in this paper, our interest lies in how teachers used expressive instructions in the classroom. That is, we focus on their creative, improvised interpretation of such texts (see for example Box 1 & Box 2 of the online Electronic Supplementary Material, which guide the teaching of the respiratory and gynaecological exam).

Before outlining our methods, a few further words of definition, particularly what we mean by improvisation and creativity, as played out in expressive instructions. From the dictionary, improvisation is defined as making or producing something from whatever is available [13]. According to Tim Ingold, it is a way of describing cultural practices more generally, a way of ‘working things out as [life] goes along’ [14]. With his collaborator Elizabeth Hallam, he argues against a limited understanding of creativity as pure innovation, a position they argue as a modernist impulse to assess the originality of end products on a scale of creativeness. Hallam and Ingold argue that this fails to acknowledge the ongoing improvisational practices of life, where there ‘is no script’ [15]. In this article, we also use these anthropological notions of creativity and improvisation to guide our analysis.

The research question of the ethnographic research upon which this article is based is: how do teachers teach, and students learn, the sensory skills of physical examination? The paper is based on two periods of ethnographic fieldwork conducted by the first author at a medical school in Maastricht, the Netherlands, during two consecutive periods over 5 years. At one stage, comparative fieldwork was also conducted by the first author in Melbourne, Australia (for findings from the Melbourne fieldwork see [16]). With this paper we focus on the Maastricht findings that concern the role of creativity and improvisation in teaching physical skills to medical students, evident through the expressive instructions of teachers. By investigating this in more detail, we argue that we learn more about the tacit components of expertise development. As the medical ethnographer Jessica Mesman argues, it is important to look for innovation within institutions, which she calls ‘exnovation’, and to look at the bodily, material practices of practitioners [17]. The method of ethnography is an excellent means by which to study such taken-for-granted practices in medical schools.

## Methods

### Methodological framework

Ethnographic research allowed a close examination of the everyday practices entailed in teaching physical skills, including both informal and formal learning encounters. By adopting this anthropological method, the authors were assuming multiple, subjective realities rather than a single objective truth, working within a long tradition of using ethnographic methods to understand medical education (see [18, 19]).

### Data collection

For this study, the first author, a medical graduate and anthropologist, conducted participant observation at a medical school in Maastricht in 2013, and later in 2017–2018, part of longer term engagement she has at this site. She was not known to the participants of this study prior to undertaking the research and did not undertake any teaching activities alongside this research. The second author, a doctor with a senior role in the medical school, facilitated entrée into these settings and collaborated during the fieldwork. He was not involved in recruitment, nor did he undertake any teaching of these students. In conducting the project, both authors were mindful that, as is the case with all research whether qualitative or quantitative, their teaching experiences and clinical backgrounds, chosen methodologies and points of access, research instruments and theoretical influences shaped all stages of the research itself [20].

All observations were undertaken at the ‘Skills Lab’, a teaching department in the medical faculty specifically designed for teaching medical students clinical skills, including those of physical examination. While the Skills Lab changed location between the different periods of fieldwork, the general classroom format largely stayed the same. Each of the Skills Lab rooms dedicated to skills learning was ‘set-up’ in a standard way according to a lesson plan. In the older Skills Lab a series of photographs on the walls indicated how tables should be arranged, how the ‘beds’ should be positioned and other equipment laid out. Each room also had a cupboard with a standard stock of tools that students used during the lesson. There were also whiteboards and projectors for PowerPoint (in the older Skills Lab) or smartboards (in the newer Skills Lab), and cloth curtains that students could use for privacy.

The content of a lesson was described in a locally-produced workbook, which was supplemented with national and international textbooks and other material. The first author observed lessons for first, second and third year students, taught in both Dutch and English, which focused on teaching different physical skills. As each year group was too big for the small class sizes at the Skills Lab, the same lesson was repeated multiple times. This meant that multiple versions of the same lesson, taught by different teachers, were observed. In this article we use examples from respiratory and gynaecological examination teaching. The basic steps taught in the practical lessons, as detailed in the workbooks, are outlined in Box 1 and Box 2 in the online Electronic Supplementary Material. The tutorials lasted 1.5 h. The purpose of the lessons was to teach students how to conduct either a) a respiratory examination or b) a vaginal examination. The respiratory examination focused on the skills of inspection, palpation, percussion and auscultation and learning to feel, to listen and to see, their own and the bodies of classmates. Many sensory skills were also taught in the vaginal examination training, although using mannequins instead.

After approaching all teachers involved in teaching physical skills at the Skills Lab department through emails, in meetings or in person to participate in the study, the first author met with interested teachers to explain the purpose of the study. At the same time, all students were also emailed about the study. Informed consent was then obtained from the students in the teaching sessions of those teachers who had consented to be part of the study. All students in each tutorial observed gave informed consent to be part of the study. No names of students were recorded, only their signatures obtained.

Over 230 h of fieldwork has been conducted to date in the Skills Lab, including 34 tutorials of 1.5 h duration, with over 500 students. This article reports on the first author’s observations of small group tutorials regarding respiratory and gynaecological examination teaching. The tutorials were taught by 11 different teachers (some taught more than once). All teachers were physicians who were or had been active in patient care. Participant observation was supplemented by informal interviews with teachers and students when possible, and study of the teachers’ and students’ texts and other resources available to them for learning.

During fieldwork, the first author made fieldnotes [21] in a notebook by hand and recorded any images drawn by teachers on the whiteboards/smartboards as well. No names were recorded in the notes, no photographs of students or teachers taken, all notes were anonymised. Handwritten fieldnotes were immediately transcribed into more detailed fieldnotes during the evening after a day of fieldwork. All handwritten notes and files were stored securely.

### Data analysis

The first author undertook the first stage of analysis after each period of fieldwork had finished, immersing herself in the material by hand-coding the transcripts for emerging themes. Interpretive notes were also made by hand on the transcripts. This thematic analysis process, an iterative process of unearthing themes in a text at different levels [22], was repeated, working back and forth across the data and relevant theoretical and empirical literature. Preliminary results of the observations of the respiratory exam teaching were presented to a group of teachers (*n* = 15) in the Skills Lab for feedback and discussion and themes were adjusted. With this first set of data, both authors engaged in further thematic analysis, discussing and verifying the important themes, themes which were then explored by the first author in analysis of the second set of data. In writing up the findings the authors have followed the Standards for Reporting Qualitative Research (SRQR) guidelines [23].

### Ethics

The project had formal ethics approval from the Dutch Medical Education Research Ethics Committee (NVMO) (Approval no. 888 and 293) and the Ethical Review Committee Inner City Faculties (ERCIC) at Maastricht University (Approval no. 030_01_03_2017).

## Results

The ethnographic thematic analysis resulted in two major themes: improvising with one’s own body and improvising with the materials at hand. We consider both to be forms of expressive instruction. These ‘techniques’ were often used by teachers simultaneously, although are presented here separately for clarity. All the observed teachers improvised to some extent using one or both of these techniques.

### Improvising with bodies

In the respiratory classes, teachers and students’ own bodies were used continually during the lesson, not only as analytical objects being percussed or auscultated, but also as sources of sounds and other sensations that could be compared with what students were required to find. An excellent example is that of vocal mimicry. Two teachers in particular used this technique, mimicking sounds, from respiratory diseases such as asthma, to resonant and dull percussion sounds, although all teachers mimicked respiratory sounds such as wheezing and stridor to some degree. Three different teachers used mimicry in another way, by showing students how they could listen with the stethoscope to their own trachea, and how this simulated bronchial breathing.

All teachers used their own bodies to demonstrate percussion techniques. During one conversation in the coffee room, during a break from teaching, the teachers compared the different techniques they taught the students. One teacher did not like ‘going up and down’ as you could not hear the differences between lung borders that way. Another used three fingers and percussed down each, one at a time. They had all learned how to percuss in different Dutch medical schools and brought these histories into their own teaching practices.

**Fig. 1:**
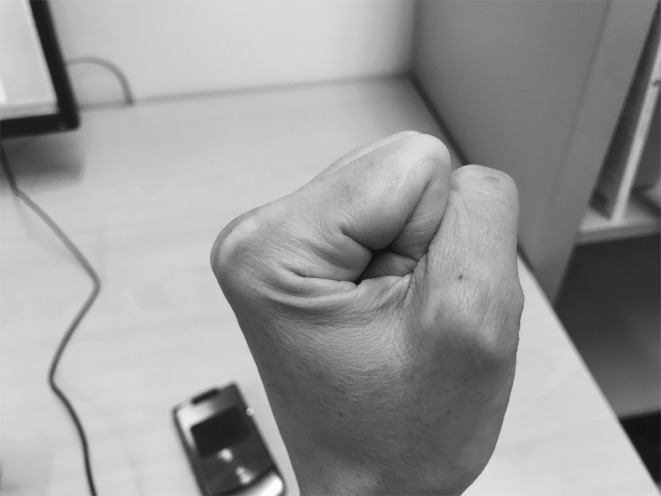
Simulated cervix

In the gynaecology examination classes, teachers used their hands throughout the lessons, not only to demonstrate techniques such as bimanual examination, or how to use a speculum, but also to simulate different parts of the body that the students were examining. Fingers were fluttered as fimbria reaching out to ovaries for example, demonstrating the anatomical structure of the female reproductive system. On another occasion a teacher scrunched her hands into a fist (see Fig. 1), making sure that there was only a small opening. This was to show the students what a cervix might look like. The teacher then gave a student a small brush that would be used in a pap smear examination, and showed the student how to rotate the brush in the small opening in her fist.

### Improvising with the materials at hand

Teachers used materials that were supplied in the classroom for the purpose of sharing sensations and manual techniques with students. In the respiratory classes, skeletons and models were used not only for showing anatomical landmarks, but also to demonstrate techniques such as auscultation. Double stethoscopes were used by two teachers so that they could tap out the sounds with the students as they listened simultaneously, correlating what they heard. PowerPoint and the smart board were used to show pictures, sounds and videos during a lesson. The teachers also brought in their own media too. They brought in CD and cassette players and accessed their favourite online sites such as repositories with recorded sounds and YouTube. The teacher who used the cassette player had selected 10 lung sounds to play to the students and he pointed out features of these sounds as he skipped through them. A teacher who used PowerPoint also had selected sounds from YouTube to play: a pleural rub, played next to a sound of someone walking through snow.

**Fig. 2:**
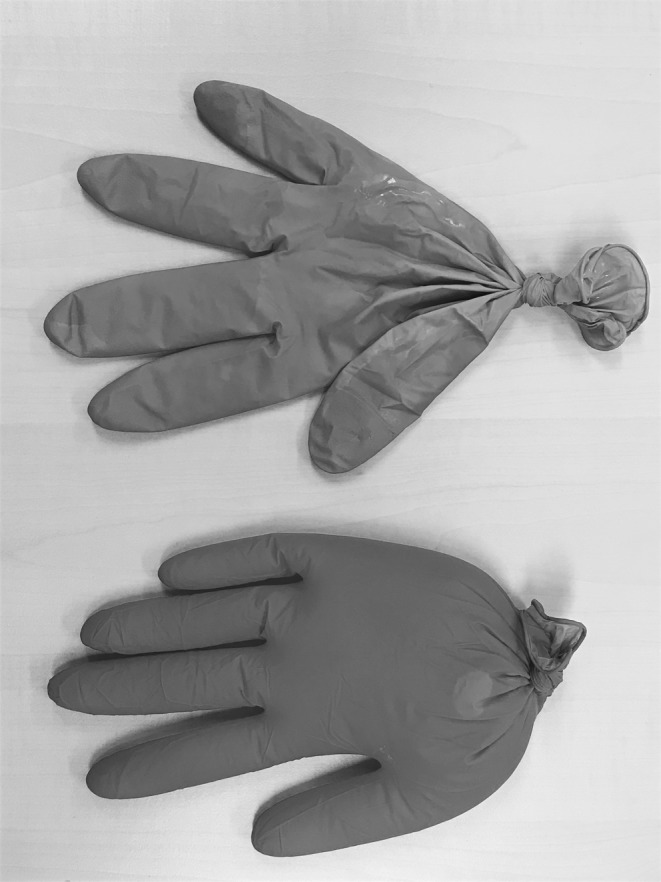
Simulated membranes

In the gynaecology examination classroom, materials were also repurposed to teach sensory skills, and in particular, to train students’ touch. A particularly creative example was the use of gloves when teaching the techniques of delivering a baby. One pair of gloves was filled with a lot of water, another with less. These were laid on the table next to another handmade teaching model: a knitted uterus in which lay a plastic baby doll. The teaching exercise proceeded as follows. The teacher pulled the baby’s head out of the cuff of the knitting, to show how the cervix widened for birth. She then inserted the glove filled with a lot of water over the baby’s head and within the knitting. A student was asked to feel the glove, and was told that this is what it felt like if the membranes had not yet ruptured. The same exercise was repeated with the glove with less water, showing what it felt like if the membranes had ruptured, but were still intact over the cervix (see Fig. 2).

These materials, either provided or brought in specifically for the lesson, did not always satisfy the teacher however, and they turned to other sources. One teacher wanted to demonstrate a heart sound and did so by pulling the privacy curtain tight. Another time the same teacher wanted to demonstrate crackles, and did so by pulling apart the Velcro on his jacket sleeves. He also demonstrated a historical technique of using what was called a pleximeter by using a tendon hammer, with a short historical lesson about percussion.

## Discussion

This ethnographic research investigates the ways in which creativity and improvisation play out in the expressive instructions used by medical educators, through the case of teaching physical skills. The results showed that teachers often had individually learned to improvise upon the basic lesson structure outlined in Box 1 and 2, by drawing creatively on their own bodies and expertise using bodily techniques of mimicry, gesture and simulation. They also used materials that were readily available and to hand, whether supplied, brought into the lesson or reinvented for a different purpose.

These expressive instructions entailed ways of explaining techniques to students by using materials that were available and working things out as the teachers went along [15]. It is notable to find that creativity and improvisation remain prevalent despite the standard Skills Lab rooms, set-ups and instruments. The push for standardization that was visible for example in the teaching protocols, assigned textbooks and photographs used to show the layout of materials for each class, did not suppress creativity. Teachers needed to find different ways to share sounds, tactility and other sensations, as this was something extremely difficult to do using the protocols only. The protocols were like recipes in that they only highlighted the main points where one might ‘get stuck‘. Between each step there is the unwritten and unsaid, the practices and experience that cannot be easily captured, and which each expert and novice approaches individually. Each teacher had his/her own approach and style and thus improvised differently with their own body and/or materials at hand. Many also incorporated clinical details of their own practice and drew upon their past experiences and expertise, in skilled ways, in order to teach their lesson.

The results show that when sharing knowledge about bodily skills in medical schools, where the material may be difficult to articulate, teachers use practices of creativity and improvisation. Many skills taught in medical school are hard to articulate, their teaching cannot be standardized, and they are often learned most significantly through repeated practice. Still, the novice must start to learn somewhere. Just like cookbook writers who use different story-telling styles to share recipes [1], the teachers in the Skills Lab chose different expressive ways to share sounds with the novices and instruct them in listening and touching. Creativity and improvisation in teaching was an integral part of training the students’ expertise and imagination. One may wonder whether the bodily improvisations found could also be collected/shared in training protocols to be used by novice teachers or whether they may already be embedded in these texts. In this article we have used examples concerning the teaching of the respiratory and gynaecological examination in medicine, but many other examples could be found elsewhere in medical education teaching. In all cases, creativity and improvisation requires great skill.

The study is limited by its focus on the classroom rather than the creation of teaching guides. Research on such material is currently being undertaken as part of longer term ethnography at the site. Also, in this article we only focus on the findings of one medical school, which could be seen as a limitation, although the Skills Lab involved is established and one of the first skills laboratories in the world, with experienced staff. An advantage of the study is that it gives unique insight into how participants were taught and learned skills through observations of the *same lesson* taught to different groups on different occasions, using the *same guide/workbook, learning goals, classroom set-ups and tools*, the difference being the teachers and the ways they used these tools. This comparability offers insights into the similarities and differences in teaching, helping to build new knowledge in dialogue with theories of craftsmanship which frame our paper.

## Further research

The findings are of educational significance for they highlight the existence and possible importance of improvisation in teaching students in medicine and training expertise, as well as the role of teachers as creative professionals [5]. As Mesman argues through her concept of exnovation, best practices are often found within existing practices, rather than new ones introduced through innovation [17]. By paying more attention to the taken-for-granted techniques of creativity and improvisation, more can be learned about how to teach the difficult, subjective experiences and techniques which are so important to making good clinical diagnoses. Further research is needed to understand more about why teachers use the techniques of improvisation that they do, to what extent their teaching practices are planned or occur ‘in the moment’ as well as their impact on students’ learning. When is improvisation appropriate or not appropriate; in what settings and teaching what subjects? In what ways are teachers’ experiences written into and reflected in protocols used in teaching? Ethnographic research offers an important way in which to understand teaching practices *in situ*; however, other methods and approaches are also needed, such as structured interviewing or textual analysis, to address some of these questions.

## Caption Electronic Supplementary Material

Box 1. Workbook lesson outline for students: The Lungs (abbreviated)

Box 2. Abbreviated Skills Lab protocols for teaching the respiratory and gynaecological examination
