# Supplementary material for: Expressive instructions: ethnographic insights into the creativity and improvisation entailed in teaching physical skills to medical students
Source: Perspect Med Educ. 2018 Jul 27;7(4):232–8. doi: 10.1007/s40037-018-0446-5 (PMC6086817; doi:10.1007/s40037-018-0446-5)
Supplement: Supplementary file 1 — Box 1. Workbook lesson outline for students: The Lungs (abbreviated) [file 40037_2018_446_MOESM1_ESM.docx]

**Expressive instructions: ethnographic insights into the creativity and improvisation entailed in teaching physical skills to medical students**

**Box 1. Workbook lesson outline for students: The Lungs (abbreviated)**

Do these assignments in pairs, but make sure you both complete all the assignments during training, so remember to swap roles in time.

1. *Inspection*
   1. *Ask your fellow student to uncover their upper body and to stand in front of you. Inspect whether healthy or ill and look for asymmetry.*
   2. *Watch the breathing of your fellow student. Count the number of breaths per minute.*
   3. *Ask your fellow student to do 10 knee bends and then assess breathing again.*
   4. *Ask your fellow student to stand and measure the circumference of the thorax. Ask student to breathe in deeply and measure the circumference again.*
2. *Palpation*
   1. *Place your hands flat on the fellow student’s thorax on left and right and feel respiratory movements.*
3. *Percussion*
   1. *Instruct your fellow student to sit on examination and determine lung border on their back. Mark borders with pencil.*
   2. *Ask student to take a deep breath in and hold it. Determine lung borders again and mark them. Ask the trainer to check the marks.*
   3. *Instruct fellow student to lie down on examination table. Percuss the area on the right of the thorax in the midclavicular line in direction of abdomen. Mark where you find the less resonant sound. Mark where you hear a dull sound. Ask the trainer to check the marks.*
   4. *Ask student to sit on the examination table. Perform comparative percussion of lungs, left and right, front and back.*
4. *Auscultation*
   1. *Take your stethoscope and use the diaphragm side to listen to the area below the clavicle on the right side. Auscultate the breathing.*
   2. *Auscultate all lung fields, front and back, left and right.*
