# Supplementary material for: Expressive instructions: ethnographic insights into the creativity and improvisation entailed in teaching physical skills to medical students
Source: Perspect Med Educ. 2018 Jul 27;7(4):232–8. doi: 10.1007/s40037-018-0446-5 (PMC6086817; doi:10.1007/s40037-018-0446-5)
Supplement: Supplementary file 2 — Box 2. Abbreviated Skills Lab protocols for teaching the respiratory and gynaecological examination [file 40037_2018_446_MOESM2_ESM.docx]

**Expressive instructions: ethnographic insights into the creativity and improvisation entailed in teaching physical skills to medical students**

**Box 2. Lesson protocol for teachers: Introduction to the Gynaecological Examination (abbreviated)**

5’      Introduction

10’    Plenary on anatomy (homework assignment has been added because the anatomy training is often scheduled after this session)

E.g. instruct the students to draw a cross-section of the small pelvis

20’     Practicing taking a gynaecological history + recording findings. e.g. play the role of patient yourself

5’      Introduction to gynaecological examination: to pay attention to:

communication (information, undressing instructions, etc.)

conditions of the examination (privacy, lighting, temperature, hygiene)

materials for the examination (gloves, kidney bowl, dressing forceps, speculum, gauze swabs, lubricant)

how to use the models (vulnerable, therefore: correct hand hygiene, short nails, no jewellery, avoid markings with pen; no gloves)

5’      Practise external inspection using the plasticised pictures

5’      Try to perform a speculum examination (disposable + Seyffert)

10’     Demonstration + explanation by the teacher:

                - speculum examination (disposable + Seyffert)

                - bimanual examination (models do not contain ovaries)

                - explain version-flexion position using model + clinical relevance

25’     In pairs, the students practice speculum examination + bimanual examination

5’      Tidying up and evaluation
